# Effects of different dual task training on dual task walking and responding brain activation in older adults with mild cognitive impairment

**DOI:** 10.1038/s41598-022-11489-x

**Published:** 2022-05-19

**Authors:** Hsiang-Tsen Kuo, Nai-Chen Yeh, Yea-Ru Yang, Wen-Chi Hsu, Ying-Yi Liao, Ray-Yau Wang

**Affiliations:** 1 Department of Physical Medicine and Rehabilitation, Taipei Chang Gung Memorial Hospital, No. 199, Tung-Hwa North Rd., Taipei, 105 Taiwan; 2 Department of Physical Therapy and Assistive Technology, National Yang Ming Chiao Tung University, No. 155, Sec. 2, Li-Nong St., Beitou Dist., Taipei, 112 Taiwan; 3 Department of Physical Medicine and Rehabilitation, Kaohsiung Municipal United Hospital, No. 976, Jhonghua 1st Rd., Gushan Dist., Kaohsiung, 804 Taiwan; 4 Department of Gerontological Health Care, National Taipei University of Nursing and Health Sciences, No. 365, Ming-Te Rd., Peitou Dist., Taipei, 112 Taiwan

**Keywords:** Geriatrics, Rehabilitation, Randomized controlled trials, Dementia

## Abstract

The concurrent additional tasking impacts the walking performance, and such impact is even greater in individuals with mild cognitive impairment (MCI) than in healthy elders. However, effective training program to improve dual task walking ability for the people with MCI is not immediately provided. Therefore, this study aimed to determine the effects of cognitive and motor dual task walking training on dual task walking performance and the responding brain changes in older people with MCI. Thirty older adults with MCI were randomly allocated to receive 24 sessions of 45-min cognitive dual task training (CDTT, n = 9), motor dual task training (MDTT, n = 11), or conventional physical therapy (CPT, n = 10). Gait performance and brain activation during single and dual task walking, and cognitive function assessed by trail-making test (TMT-A, B) and digit span test were measured at pre-, post-test, and 1-month follow-up. Both CDTT and MDTT improved dual task walking with responding activation changes in specific brain areas. The improvements in motor dual task walking performance after both dual task trainings were significantly better than after CPT in the older adults with MCI. Both cognitive and motor dual task training were feasible and beneficial to improve dual task walking ability in older adults with MCI.

**Trial Registration:** The trial was registered to Thai Clinical Trial Registry and the registration number is TCTR20180510002 (first registration date: 10/05/2018).

## Introduction

Dual task walking, such as talking or carrying a bag while walking, is essential in daily life. However, both cognitive and motor dual task impact the walking performance, including decreased speed, increased stride time and stride time variability^1–3^, especially in the older population^4^. The impaired gait performance may indicate a risk of fall^5,6^, which is an important issue for the aged populations. As dual task walking may be affected by motor ability^7^, growing evidence demonstrated that the gait performances highly correlated with general cognitive function in the healthy older population^3^. Prefrontal cortex (PFC) plays a crucial role in cognitive function, and was evident to be involved in dual tasking^8,9^. Increased activation in PFC was observed during dual task walking in healthy adults, and older adults tended to activate more compared to younger participants^10^. Other motor brain areas such as premotor cortex (PMC) and supplemental motor area (SMA) were also found to activate during dual task walking, which were responsible for stabilizing gait and adapting walking speed^11–14^.

Cognitive impairment in aging population has drawn growing attention in the recent decade. Mild cognitive impairment (MCI) is a brain function syndrome involving the onset and evolution of cognitive impairments beyond those expected based on the age and education of the individual^15^. In people with MCI, decreased speed, increased stride time, variability were observed during cognitive dual task walking as compared with single walking, with inconclusive results in PFC activation^16–21^. The influence of additional task on walking was even greater in individuals with MCI than in healthy elders^17,19,21^, which may further impact their daily activities and increase fall risks. Thus, dual task walking ability should be addressed especially in cognitive impaired population. However, the effective training program to improve dual task walking ability is not immediately provided.

Dual task training is designed in accordance with the principles of motor learning and task specificity, to improve the dual task performance^22^. The beneficial effects of dual task training have been demonstrated in several populations with different clinical conditions^23^. Specifically, cognitive dual task training has been reported to exert beneficial effects on cognitive and physical functions in older adults with and without cognitive impairment^24,25^. The overview of systematic reviews has further suggested that such training program is preferred over single task training as targeting motor performance under dual task situation in healthy older adults^24^. However, the effect of dual task training on dual task walking performance in older adults with MCI is not known. Furthermore, whether different dual task training would exert different effects in the MCI population needs further elucidation for possible clinical applications. Therefore, this study aimed to determine the effects of cognitive and motor dual task walking training on dual task walking performance and responding brain changes in older adults with MCI. We hypothesized that cognitive and motor dual task walking training would provide task-specific effects on dual task walking performance in older adults with MCI, and the brain changes would correlate to the improvement of walking performance after training.

## Results

**Figure 1:**
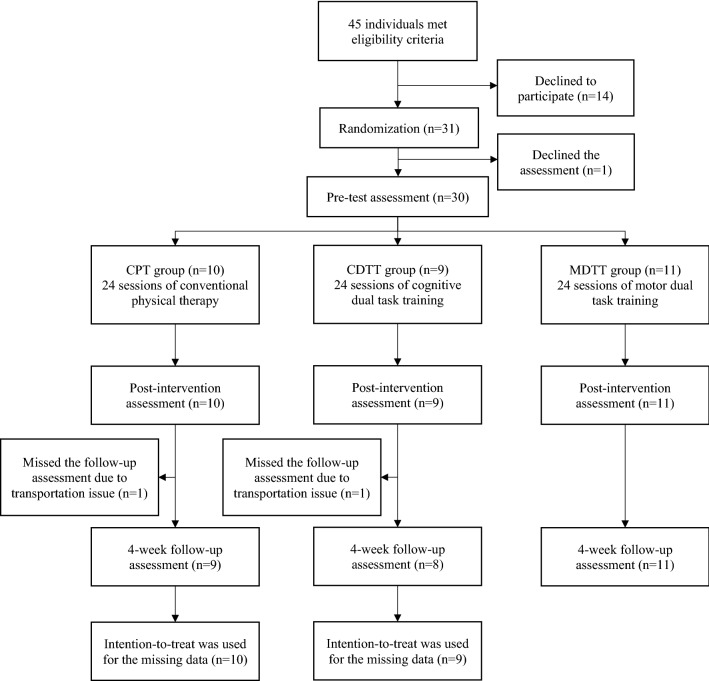
Study flow chart. *CPT* conventional physical therapy, *CDTT* cognitive dual task training, *MDTT* motor dual task training.

Forty-five older adults with MCI who met the eligibility criteria were recruited, and 14 of them declined to participate due to personal reasons. Thirty-one participants were randomly assigned to the conventional physical therapy group (CPT, n = 10), cognitive dual task training group (CDTT, n = 10), or motor dual task training group (MDTT, n = 11). One in the CDTT group dropped out before the pre-test assessment. Therefore, total of 30 participants completed the training sessions and post-test measurements. One in the CPT group and one in the CDTT group missed the follow-up assessment because of transportation issue. The study flow chart was shown in Fig. 1.

The median age for all participants was 79.5 (interquartile range, IQR = 74.3–85.3) years old. The median mini-mental state examination (MMSE) scores were 28 (IQR = 27–29) and the median Montreal cognitive assessment (MoCA) scores were 22 (IQR = 20–23.8). No significant differences among groups were found in basic data and measures at pre-test (Table 1).

**Table 1:** Basic data of participants.

	Conventional physical therapy group (n = 10)	Cognitive dual task training group (n = 9)	Motor dual task training group (n = 11)	p value
Age, y	79 (71, 86.8)	80 (76.5, 86)	78 (70, 81)	0.282
Gender, male: female	5:5	1:8	1:10	0.050
MMSE	28.5 (27.8, 29.3)	28.0 (27.5, 29)	28.0 (26, 30)	0.688
MoCA	22.0 (20, 25)	22.0 (21, 24)	22.0 (20, 23)	0.798
Educational level, y	10.5 (6, 12)	12.0 (9, 12)	9.0 (6, 12)	0.280

Values are median (interquartile) or frequency.

*MMSE* Mini Mental State Examination, *MoCA* Montreal Cognitive Assessment.

### Cognitive dual task walking

Table 2 shows the results of cognitive dual task walking performance after different interventions. The CDTT resulted in significant within-group improvements in speed, cadence, stride length, and stride time. The improvement in stride length correlated with the increase in PFC (rho = 0.850, p = 0.007), bilateral PMC (left PMC: rho = 0.778, p = 0.023; right PMC: rho = 0.838, p = 0.009) and SMA (rho = 0.910, p = 0.002) after CDTT. Significant within-group training effects of MDTT were observed on all the gait parameters, except for temporal variability. However, the improvement in gait performance after MDTT did not correlate significantly with the change in brain activation. There was no significant difference across time in the CPT group. The training effects on speed, stride length, and spatial variability were significantly better after MDTT than CPT at follow-up.

**Table 2:** Comparisons of cognitive dual task walking performance after training at different time point.

	Conventional physical therapy group (n = 10)				Cognitive dual task training group (n = 9)				Motor dual task training group (n = 11)				Between-group comparisons	
	Pre	Post	Follow-up	p^a^	Pre	Post	Follow-up	p^a^	Pre	Post	Follow-up	p^a^	Post	Follow-up
													p (ε^2^)	p (ε^2^)
Speed (cm/s)	72.3 (58, 89.2)	79.3 (61.4, 96.5)	79.3 (51.8, 93.9)	0.122	63.9 (42, 87.9)	69.5 (50.3, 96)	72.0 (52, 101.3)	0.004	71.8 (62.8, 81.3)	81.8 (63.3, 89.8)	89.2 (81.8, 96)	0.009		
Change		7.2 (2.3, 14.1)	4.5 (− 11, 11.9)			8.2 (7.6, 11.6)	9.0 (4.0, 16.0)			17.0 (0.3, 19.3)	18.1 (12.3, 27.3)*		0.594 (0.036)	0.020 (0.264)
Cadence (step/min)	104.8 (95.9, 109)	106.5 (98.6, 114.7)	107.3 (96.6, 116.4)	0.202	100.6 (73.6, 107.5)	105.0 (81, 113.1)	103.3 (87.5, 117.6)	0.016	104.0 (99.1, 107.9)	106.5 (102.4, 113.7)	112.2 (107.5, 114.7)	0.020		
Change		4.4 (0.6, 5.3)	3.3 (− 4.5, 9.5)			6.2 (3.4, 11.5)	8.6 (3.1, 13.7)			5.9 (0.6, 11.1)	6.4 (2.4, 15.6)		0.412 (0.061)	0.196 (0.113)
Stride length (cm)	86.9 (70.7, 103)	94.6 (70.2, 104.6)	90.1 (67.3, 105.1)	0.150	71.8 (67.1, 97.6)	76.0 (71.8, 101.3)	81.3 (70.8, 103.5)	0.016	82.3 (75, 90.3)	90.0 (73, 99.8)	95.7 (87, 107)	0.020		
Change		5.5 (1.2, 10.8)	0.4 (− 9.6, 7.8)			5.5 (1.8, 7.4)	4.9 (1.8, 7.8)			9.7 (− 1.8, 12.5)	13.5 (8.3, 23.6)*		0.458 (0.054)	0.005 (0.357)
Spatial variability	7.2 (5.3, 11.1)	7.3 (5.5, 10.1)	7.3 (5.2, 12.2)	0.202	9.5 (7.1, 12)	9.0 (6.4, 11.2)	8.0 (7.2, 9.6)	0.459	9.3 (7.2, 11.7)	8.5 (6.9, 12.1)	6.9 (5.5, 8.0)	0.020		
Change		− 0.1 (− 1.4, 0.9)	0.9 (− 1.3, 1.8)			− 1.4 (− 2.6, 2.4)	− 2.0 (− 2.9, 0.1)			− 1.0 (− 2.3, 1.4)	− 1.9 (− 4.8, − 0.9)*		0.841 (0.012)	0.014 (0.294)
Stride time (s)	1.2 (1.1, 1.3)	1.1 (1.0, 1.2)	1.1 (1.0, 1.2)	0.232	1.2 (1.1, 1.7)	1.1 (1.1, 1.5)	1.2 (1.0, 1.4)	0.015	1.2 (1.1, 1.2)	1.1 (1.1, 1.2)	1.1 (1.0, 1.1)	0.006		
Change		− 0.1 (− 0.1, 0)	0.0 (− 0.1, 0.1)			− 0.1 (− 0.2, − 0.1)	− 0.1 (− 0.2, − 0.1)			− 0.1 (− 0.1, 0)	− 0.1 (− 0.2, 0)		0.243 (0.100)	0.091 (0.165)
Temporal variability	5.4 (2.6, 7.9)	4.6 (3.0, 5.5)	4.0 (2.8, 9.2)	0.670	7.1 (4.0, 10.1)	4.9 (3.5, 7.2)	5.7 (4.8, 7.1)	0.121	7.3 (4.5, 15.3)	6.4 (5.1, 13)	6.6 (5.3, 7.2)	0.078		
Change		− 1.0 (− 2.8, 0)	0.0 (− 1.6, 0.9)			− 1.9 (− 2.7, − 0.5)	− 0.5 (− 3.7, 1.2)			− 0.5 (− 2.0, 1.2)	− 1.5 (− 8.6, 1.0)		0.361 (0.070)	0.528 (0.044)
DTC (%)	19.7 (23, 7.2)	16.2 (23, 7.4)	16.1 (26.8, 9.1)	0.301	20.0 (28.6, 4.9)	21.9 (33.6, 8.9)	21.8 (33.9, 6.9)	0.641	16.6 (22.1, 11.9)	19.3 (24.9, 14.8)	10.1 (15.5, 5.4)	0.020		
Change		1.3 (− 7.9, 5.4)	3.4 (− 6.5, 9.0)			− 0.5 (− 2.2, 6.7)	2.2 (− 4.5, 7.9)			5.1 (− 1.3, 5.9)	− 6.5 (− 10.7, − 0.3)		0.483 (0.050)	0.063 (0.190)

Values are median (interquartile).

Change values were calculated by subtracting the baseline data from the post-training data (Post) or by subtracting the baseline data from the follow-up data (Follow-up).

^a^p value for within-group comparison using Friedman test.

*p < 0.05 as compared with conventional physical therapy group.

### Motor dual task walking

The motor dual task gait performance in three groups are presented in Table 3. The significant improvements in speed, cadence, stride length, stride time, spatial variability, and dual task cost (DTC) of gait speed were found in the MDTT group. Only the change in spatial variability correlated with PFC after MDTT (rho = 0.857, p = 0.007). On the other hand, CDTT also provided beneficial effects on cadence and stride time during motor dual task walking. The improvement in cadence correlated with PFC (rho = 0.810, p = 0.015) and right PMC (rho = 0.714, p = 0.047), and the improvement in stride time correlated with PFC (rho = − 0.874, p = 0.005) and right PMC (rho = − 0.934, p = 0.001) after CDTT. No significant improvement was shown in the CPT group. The between-group differences among three groups were found significant at both post-test and follow-up assessment. Both MDTT and CDTT provided greater improvements on cadence and stride time than CPT at the end of the training programs. The improvement in cadence remained for 4 weeks after CDTT, while the changes in speed, stride length and spatial variability in the MDTT group were significantly greater than that in the CPT group at follow-up.

**Table 3:** Comparisons of motor dual task walking performance after training at different time point.

	Conventional physical therapy group (n = 10)				Cognitive dual task training group (n = 9)				Motor dual task training group (n = 11)				Between-group comparisons	
	Pre	Post	Follow-up	p^a^	Pre	Post	Follow-up	p^a^	Pre	Post	Follow-up	p^a^	Post	Follow-up
													p (ε^2^)	p (ε^2^)
Speed (cm/s)	70.4 (64.1, 100.7)	69.0 (64.5, 94.2)	70.0 (62, 100.1)	0.670	72.8 (55.8, 86.6)	71.3 (62.1, 79.8)	74.9 (69.3, 91.1)	0.459	77.0 (64, 93)	89.4 (78, 100)	98.0 (80.3, 104)	0.003		
Change		− 0.1 (− 7.1, 6.4)	0.1 (− 5.8, 5.1)			− 0.5 (− 12, 10)	4.8 (− 5.6, 12.1)			12.4 (7.5, 21.3)	17.3 (11.3, 27)*		0.028 (0.246)	0.006 (0.352)
Cadence (step/min)	105.9 (98.4, 116.5)	102.6 (96.9, 115.9)	100.2 (90.8, 117)	0.741	103.5 (94.9, 110.6)	108.2 (100.8, 117.5)	112.4 (103, 121.3)	0.008	108.2 (100.8, 115.7)	116.4 (108.1, 123.3)	114.8 (108.3, 120.3)	0.004		
Change		− 1.5 (− 5.4, 2.1)	− 3.5 (− 7.4, 2.4)			5.8 (2.5, 7.0)*	8.0 (1.0, 13.7)*			3.9 (1.5, 9.0)*	4.8 (1.1, 10.9)		0.007 (0.346)	0.017 (0.280)
Stride length (cm)	86.9 (72.1, 112.4)	86.0 (75.8, 105.3)	88.9 (75.3, 112.3)	0.387	80.5 (73, 91.3)	79.2 (68.1, 88.3)	82.3 (73.4, 92.3)	0.641	85.6 (71.8, 97.3)	94.2 (83.3, 104)	103.0 (85.3, 112.8)	0.003		
Change		1.0 (− 1.4, 8.7)	0.8 (− 4.8, 3.0)			− 4.3 (− 11.3, 3.4)	2.0 (− 8.9, 6.9)			8.6 (6.3, 15)^#^	13.3 (6.0, 25.3)*^,#^		0.033 (0.235)	0.007 (0.345)
Spatial variability	6.7 (5.0, 9.5)	7.9 (4.5, 8.8)	6.9 (4.7, 8.8)	0.905	8.1 (5.8, 9.5)	6.8 (6.6, 11.4)	7.4 (6.4, 8.6)	0.236	7.4 (6.3, 8.7)	7.1 (5.9, 7.6)	5.5 (4.1, 6.9)	0.012		
Change		− 0.3 (− 0.6, 0.9)	0.0 (− 0.8, 0.4)			0.8 (− 0.5, 2.0)	− 0.8 (− 1.0, 0.7)			0.1 (− 2.4, 0.5)	− 2.0 (− 3.5, − 0.6)*		0.150 (0.131)	0.013 (0.302)
Stride time (s)	1.1 (1.0, 1.2)	1.2 (1.1, 1.3)	1.1 (1.0, 1.3)	0.301	1.2 (1.1, 1.3)	1.1 (1.0, 1.2)	1.1 (1.0, 1.2)	0.013	1.1 (1.0, 1.2)	1.1 (1.0, 1.1)	1.1 (1.0, 1.1)	0.012		
Change		0.0 (0, 0.2)	0.0 (0, 0.1)			− 0.1 (− 0.1, 0)*	− 0.1 (− 0.2, 0)*			0.0 (− 0.1,0)*	0.0 (− 0.1, 0)		0.003 (0.399)	0.039 (0.223)
Temporal variability	4.3 (3.2, 5.5)	4.4 (3.3, 4.9)	3.5 (2.8, 6.1)	1.00	3.3 (3.0, 7.4)	3.8 (3.3, 6.4)	3.9 (3.4, 5.3)	0.459	5.3 (3.7, 7.8)	5.0 (3.6, 7.2)	4.5 (3.7, 5.3)	0.060		
Change		− 0.1 (− 0.9, 1.5)	0.1 (− 1.0, 1.0)			− 0.2 (− 1.0, 0.7)	0.3 (− 1.0, 0.7)			− 0.3 (− 1.0, 0.5)	− 1.0 (− 2.7, − 0.1)		0.672 (0.027)	0.160 (0.126)
DTC (%)	9.4 (17, 1.7)	18.3 (21.1, 4.1)	16.5 (20.1, 3.3)	0.273	5.7 (9.4, 3.5)	19.3 (32.3, 7.9)	15.8 (23.8, 6.7)	0.008	6.6 (19.8, 4.0)	9.7 (16.9, 6.5)	5.9 (9.5, 1.7)	0.020		
Change		4.7 (− 2.0, 10.8)	4.6 (− 2.8, 9.0)			13.3 (5.1, 23)	7.6 (4.2, 16.9)			2.9 (− 1.3, 6.1)	− 2.6 (− 7.8, − 1.0)^#^		0.056 (0.199)	0.008 (0.335)

Values are median (interquartile).

Change values were calculated by subtracting the baseline data from the post-training data (Post) or by subtracting the baseline data from the follow-up data (Follow-up).

^a^p value for within-group comparison using Friedman test.

*p < 0.05 as compared with conventional physical therapy group.

^#^p < 0.05 as compared with cognitive dual task training group.

### Single walking

The speed, cadence, stride length, and stride time during single walking were improved after CDTT. Significant within-group improvements in speed, cadence, stride length, spatial variability, and stride time were found in the MDTT group. No significant change was found after CPT. Furthermore, the training effects on cadence and stride time after CDTT was significantly better than CPT at follow-up (Table 4). However, the improvements in gait performance did not significantly correlate with the change in brain activation after the cognitive or motor dual task training.

**Table 4:** Comparisons of single walking performance after training at different time point.

	Conventional physical therapy group (n = 10)				Cognitive dual task training group (n = 9)				Motor dual task training group (n = 11)				Between-group comparisons	
	Pre	Post	Follow-up	p^a^	Pre	Post	Follow-up	p^a^	Pre	Post	Follow-up	p^a^	Post	Follow-up
													p (ε^2^)	p (ε^2^)
Speed (cm/s)	86.6 (68.4, 112.2)	91.9 (78.5, 114.8)	93.1 (75.9, 110.1)	0.387	77.5 (60.6, 88.3)	90.3 (76.3, 100)	92.6 (74.2, 108.3)	0.005	84.8 (73, 95.8)	99.4 (84.3, 110.8)	99.3 (88.3, 109.5)	0.035		
Change		8.3 (− 4.4, 14.8)	3.3 (− 2.6, 12.8)			12.8 (7.5, 18.5)	15.1 (12.8, 21)			14.9 (10.3, 27.8)	16.8 (3.0, 24.7)		0.129 (0.141)	0.036 (0.225)
Cadence (step/min)	106.5 (103.3, 116.6)	109.8 (102.4, 117.7)	111.7 (101.5, 120.9)	0.497	103.5 (97.2, 111.1)	112.2 (102, 122.1)	113.7 (107.2, 122)	0.016	109.2 (98.3, 113.7)	116.9 (114.8, 121.3)	116.3 (111.4, 122.3)	0.035		
Change		− 0.2 (− 2.5, 6.4)	2.0 (− 1.4, 6.6)			8.7 (4.2, 12.8)	10.2 (7.2, 14.7)*			6.4 (2.6, 10.8)	5.8 (1.9, 9.5		0.075 (0.179)	0.032 (0.237)
Stride length (cm)	98.0 (80.1, 117.9)	105.8 (88.3, 116.4)	102.6 (87.9, 119.3)	0.273	84.0 (77.8, 98.3)	91.8 (86.8, 103.1)	93.5 (86.8, 108.3)	0.013	89.5 (83, 101)	102.6 (86, 116.8)	102.6 (91, 117.3)	0.050		
Change		5.4 (− 1.6, 14.9)	1.1 (− 1.3, 6.4)			6.4 (1.3, 10.9)	10.0 (3.8, 10.3)			10.5 (3.8, 15.8)	8.0 (0, 22)		0.547 (0.040)	0.104 (0.156)
Spatial variability	6.2 (5.0, 9.8)	5.9 (4.7, 7.4)	5.3 (4.3, 7.5)	0.407	6.8 (5.0, 9.2)	6.1 (5.6, 6.9)	6.4 (5.9, 6.9)	0.641	7.4 (6.2, 8.1)	6.5 (4.8, 8.9)	5.9 (4.6, 6.7)	0.020		
Change		− 0.7 (− 2.0, 0.4)	− 0.7 (− 1.7, 0.4)			− 0.3 (− 1.3, 1.4)	− 0.4 (− 1.8, 1.4)			− 0.6 (− 1.7, 1.5)	− 1.1 (− 1.9, − 0.4)		0.728 (0.022)	0.353 (0.072)
Stride time (s)	1.1 (1.0, 1.2)	1.1 (1.0, 1.2)	1.1 (1.0, 1.2)	0.497	1.2 (1.1, 1.3)	1.1 (1.0, 1.2)	1.1 (1.0, 1.1)	0.013	1.1 (1.1, 1.2)	1.0 (1.0, 1.0)	1.0 (1.0, 1.1)	0.029		
Change		0.0 (− 0.1, 0)	0.0 (0, 0)			− 0.1 (− 0.2, 0)	− 0.1 (− 0.2, − 0.1)*			− 0.1 (− 0.1, 0)	− 0.1 (− 0.1, 0)		0.132 (0.141)	0.024 (0.256)
Temporal variability	3.0 (2.3, 4.0)	3.3 (2.6, 4.0)	3.6 (2.3, 4.2)	0.717	4.0 (3.2, 6.4)	3.4 (3.0, 4.8)	3.3 (2.8, 4.0)	0.097	4.9 (4.0, 6.1)	4.3 (3.0, 5.4)	3.6 (3.3, 4.2)	0.086		
Change		0.2 (− 0.6, 0.7)	0.0 (− 0.1, 0.7)			− 0.5 (− 1.8, 0.4)	− 1.3 (− 2.8, − 0.1)			− 0.6 (− 1.3, 0.7)	− 1.1 (− 2.0, − 0.3)		0.436 (0.057)	0.075 (0.179)

Values are median (interquartile).

Change values were calculated by subtracting the baseline data from the post-training data (Post) or by subtracting the baseline data from the follow-up data (Follow-up).

^a^p value for within-group comparison using Friedman test.

*p < 0.05 as compared with conventional physical therapy group.

### Cognitive function

There were no significant changes in cognitive function after CDTT, MDTT or CPT, except for the trail-making test part B (TMT-B). The time needed to complete TMT-B was significantly less after 24 sessions of MDTT, and such effect remained at the 4-week follow-up (Table 5).

**Table 5:** Comparisons of cognitive function after training at different time point.

	Conventional physical therapy group (n = 10)				Cognitive dual task training group (n = 9)				Motor dual task training group (n = 11)				Between-group comparisons	
	Pre	Post	Follow-up	p^a^	Pre	Post	Follow-up	p^a^	Pre	Post	Follow-up	p^a^	Post	Follow-up
													p (ε^2^)	p (ε^2^)
TMT-A	22.1 (11.5, 32.4)	13.9 (9.2, 18.8)	17.4 (8.2, 21.7)	0.122	21.9 (17.7, 24.1)	22.9 (14.1, 27.1)	22.9 (15.9, 31.8)	1.00	21.2 (10.9, 36.8)	12.5 (8.5, 18.8)	15.3 (10.6, 20.1)	0.061		
Change (%)		− 26.1 (− 49.3, 20.1)	− 25.2 (− 40.0, − 6.3)			0.48 (− 46.1, 12.5)	− 3.34 (− 34.9, 38.8)			− 34.3 (− 48.7, − 19.7)	− 33.1 (− 44.4, 34.4)		0.453 (0.055)	0.627 (0.035)
TMT-B	58.2 (32.5, 78.3)	50.6 (31.6, 75.9)	55.0 (25.5, 75.2)	0.670	40.3 (35.9, 107.9)	61.4 (35.4, 87.6)	63.6 (30.8, 72.0)	0.417	67.8 (41.6, 76.1)	46.8 (36.7, 88.7)*	39.7 (25.6, 55.6)*	0.001		
Change (%)		− 10.3 (− 35.1, 16.0)	− 14.5 (− 28.6, 27.8)			1.02 (− 30.1, 27.5)	− 23.0 (− 46.5, − 0.01)			− 23.9 (− 45.8, − 6.9)	− 22.0 (− 52.6, − 14.9)		0.357 (0.071)	0.210 (0.116)
Digit span test	13.0 (11, 18.8)	16.0 (14.8, 19.3)	16.0 (13.5, 20.3)	0.063	18.0 (13, 20)	19.0 (17, 23)	15.0 (12.3, 19.5)	0.114	14.0 (11, 18)	16.0 (11, 20)	16.5 (11, 21)	0.641		
Change (%)		16.8 (4.17, 32.2)	10.1 (− 2.17, 32.2)			30.8 (− 2.08, 49.7)	0 (− 18.6, 27.1)			10.0 (− 6.67, 27.3)	2.94 (− 5.53, 11.7)		0.316 (0.079)	0.568 (0.042)

Values are median (interquartile).

Change values were calculated by (post data or follow-up data − pre data) / pre data × 100%

*TMT* Trail-Making Test.

^a^p value for within-group comparison using Friedman test.

*p < 0.05 for within group difference compared to pre-test.

## Discussion

Although many studies have examined the interventions on cognitive function in individuals with MCI^26–28^, few have explored the effect of different training on gait performance^29^. This study demonstrated both cognitive and motor dual task trainings improved dual task walking and single walking performance. Furthermore, the effects of both dual task trainings on the motor dual task walking performance were superior to CPT in the older adults with MCI. Nevertheless, only MDTT resulted in significantly superior effect at 1-month on both dual task walking performances as compared with CPT. In addition, improvements in dual task gait performance correlated with the activations in the specific brain areas.

As dual task walking ability is essential for daily activities, previous studies have shown that simultaneous cognitive and aerobic training improved cognitive dual task walking ability more than physical training in people with MCI^30^. However, the virtual reality-based dual task balance training did not benefit the Timed Up-and-Go test under dual task condition in the institutionalized older persons with MCI^31^. In present study, both cognitive and motor dual task trainings improved walking performance under dual task conditions, but the CPT, as practicing single tasks consecutively, did not. Present results were consistent with results of other studies in different populations^32,33^, suggesting the specific dual task training effect over single task training. Moreover, the improvements in gait speed (median: 18.1 and 17.3 cm/s for cognitive and motor dual task walking, respectively) were higher than the minimum detectable change value (16 cm/s) during dual task walking^34^, which may indicate the clinical importance of the favorable results.

It draws our attention that motor and cognitive dual task training did not result in distinct effects in accordance with the dual task types in the MCI population. Previously we found that CDTT improved cognitive dual task gait performance and MDTT improved motor dual task gait performance in people with chronic stroke^35^. The reasons for inconsistent results between participants with MCI and stroke are not known. We further noted the lasting effects (at 1-month follow-up) on dual task walking performance seemed to be evident after MDTT, while the lasting effects on single walking performance was significant after CDTT. The concept of task priority may lend some explanations for such results after motor and cognitive dual task training. Previous studies proposed the prioritization of dual task performance depended on the complexity of the posture-related task as well as the additional tasks^36–38^. In present study, the additional cognitive tasks may be too challenging for our participants to maintain the posture control and gait stability, thus they needed to allocate attentional resources on posture and gait control during CDTT. As a result, we observed the significant improvement in single walking performance after CDTT with little changes in cognitive function tested by TMT and digit span test. The insignificant improvements in cognitive function as demonstrated by executive and memory function were also noted in the meta-analysis study after combined cognitive-physical intervention^39^.

On the other hand, the additional motor tasks did not seem very complex or difficult, so the participants may not need to prioritize the additional task over the walking during MDTT. In other words, MDTT might benefit from minimizing the crosstalk, which reduced the requirement for attention on the additional motor tasks during training^40^. Therefore, the participants could have ample opportunity to practice the integration of two simultaneous tasks, which resulted in superior beneficial effects on dual task walking performance even after 1-month post training. In addition, it should be mentioned the implications of changes in gait variability after MDTT. Studies showed that the gait variability was able to predict fall risk, and was related to executive function in the aging populations with and without cognitive impairment^41^. In line with the improvement in dual task walking performance, we noticed the beneficial effects on TMT-B after MDTT. As mentioned earlier, cognitive flexibility plays an important role in dual tasking in the aged population^42^, and TMT-B is used to assess executive function, specifically as the ability of cognitive flexibility^43^. Taking together, our MDTT program improved dual task walking ability by the adaptation of dual tasking and possibly decreased fall risks in older adults with MCI.

It is worth mentioning that our two dual task training programs were also able to exert clinically important improvements in single walking speed, as the median change values (15.1 and 16.8 cm/s at follow-up in CDTT and MDTT group, respectively) were in the range of minimal clinical important differences (10–20 cm/s)^44^. By contrast, CPT did not improve any walking performance measured in present study. A meta-analysis demonstrated multi-modal training, similar to CPT in present study, could improve single walking performance with a relatively long period of training, e.g. 90 min per session for 40 sessions in older adults with MCI or mild dementia^45^. Our CPT programs were 45 min per session, including 15 min of gait training, for a total of 24 sessions. On the other hand, our dual task training combined either cognitive or motor task concurrently with gait training throughout the 45-min session, and resulted in single walking improvement. Therefore, to improve single walking performance may need relatively long duration of walking activity.

Although both cognitive and motor dual task training exerted beneficial effects on dual task walking ability, the underlying brain changes in response to different types of dual task training seemed different, which may indicate different strategies for the improvements^46^. According to our results, positive correlations between the changes in gait performance and brain activation after CDTT, suggesting that CDTT enhanced the brain activations in prefrontal and motor-associated brain areas extensively to provide positive effects. In line with our findings, previous study proposed that older adults increased the activation in SMA and frontal cortex as a compensatory strategy to ensure a better task performance^47^. PMC and SMA played important roles in gait control, and prioritizing the walking tasks during CDTT might specifically induce activations of these brain areas. Regarding the PFC activation, although it may reach the resource ceiling for cognitive tasks in the aged population, Logan et al. demonstrated that providing external instructions potentially helped to reverse the deactivation of frontal cortex^48^. Thus, we speculate that CDTT may increase the recruitment of PFC resource by verbal cues from the therapist, which may partially contribute to the improvements in walking performance. As for the MDTT, the reduction in spatial variability correlated with the decreased activation in PFC during motor dual task walking after training, which may be explained by the cortical adaptation for the processing of dual-tasking^49^. Previous studies demonstrated decreased PFC activation at late stage of motor learning, accompanied by the shifting of neural networks^50,51^ to promote neural efficiency even after the learning process^46^. We thus think the beneficial effects on dual task walking of MDTT may be related to the enhancement of neural efficiency, and that of CDTT may be related to the recruitment of neural resources.

There were several limitations in this study should be noted. First, the sample size was relatively small in each training group, and there were drop-outs at the 1-month follow-up. To address this issue, we applied the non-parametric statistical methods with Bonferroni adjustment and intention-to-treat in order to draw unbiased conclusion. The significances in the between-group comparisons were with large effect size, which may help to validate the training effects. Second, the task priority was not specifically emphasized during our dual task training programs, and the instructions were given according to the performance for each participant during training. Further studies are needed to investigate the influences of task prioritization and task complexity on training effects to establish a comprehensive guideline for the application of dual task training.

Our study provides the first evidence about different dual task trainings on dual task walking performance and related brain changes in individuals with MCI using three parallel groups with randomized controlled design. Both cognitive and motor dual task training for 24 sessions improved walking performance under single and dual task condition, while the CPT did not exert significant change in gait performance. Furthermore, the changes in responding brain activation during walking were related to dual task walking improvement after CDTT and MDTT. The possible mechanisms for the different dual task training should be investigated and validated in future studies.

## Methods

### Study design

This study was a three-arm parallel single blinded (assessor blinded), randomized controlled trial. The Block of 6 randomization was used, and the random allocation sequence was generated via sealed envelope drawn by a person who was not involved in the study. Participants were enrolled and randomly assigned by the researchers to one of the three groups: cognitive dual-task training (CDTT), motor dual-task training (MDTT) or conventional physical therapy group (CPT). A sample of 30 participants was suggested to be sufficient with effect size of 0.4 (medium effect size), power of 0.80 and a two-tailed alpha level of 0.05 using G*Power version 3.1. The basic data including age, gender, educational level, mini-mental state examination (MMSE) scores and Montreal cognitive assessment (MoCA) scores were obtained and assessed before the interventions. All participants received 45 min of training according to their group assignment, 3 sessions per week for a total of 24 sessions instructed by a physical therapist. All outcomes were measured before (pre) and after completing 24 sessions of interventions (post), and at 1-month follow-up assessment (follow-up). The study protocol was approved by the Institutional Review Board of National Yang-Ming University (approval number: YM107032F). The trial was prospectively registered to Thai Clinical Trial Registry (TCTR20180510002) on 10/05/2018 and conformed to the CONSORT checklist. All experiments were performed in accordance with relevant guidelines and regulations.

### Participants

Older adults with MCI were recruited from community centers, nursing homes and long-term care facilities in Taiwan. The inclusion criteria were (1) age ≥ 65 years old, (2) MCI was determined by MMSE ≥ 24 and MoCA < 26^52–54^, and (3) the ability to walk for 60 s independently without any assistive device. The exclusion criteria included (1) unstable medical condition and (2) any neurological disease, psychological disorder, or learning disability known to interfere with participation in the study. Informed consent was obtained from all participants.

### Outcome measures

The primary outcome was gait performance under dual task conditions, and the secondary outcomes included single walking performance, brain activation during walking, and cognitive function. Data for gait performance and brain activation were obtained simultaneously during 3 walking conditions described below. Participants were asked to sit on a chair for 60 s, and then to stand still for at least 15 s to stabilize the hemodynamic response before each walking trial. The participants were asked to walk back and forth on the hallway for 60 s in each walking trial. Each condition repeated twice with a total of 6 walking trials in a random order.

- Single walking (SW): Participants were asked to walk at their self-selected speed.
- Walking while performing a cognitive task: Participants were asked to walk at their self-selected speed while subtracting three from a random three-digit number serially.
- Walking while performing a motor task: Participants were asked to walk at their self-selected speed while carrying a tray, with a cup of water on it, with both hands.

### Gait performance

The gait performance was assessed by two wearable Physilog sensors (GaitUp system, Lausanne, Switzerland) attached to the lateral side of each shoe. The sensor was validated and had fair reliabilities (0.71–0.87) for measuring single and dual task walking performance^34,55^. The kinematic data was recorded and synchronized wirelessly for two sensors, and transferred to a tablet computer. Gait parameters included speed, cadence, stride length, stride time, and gait variability. The spatial and temporal gait variability were calculated as the coefficient of variation (= standard deviation/mean × 100%) of the stride length and stride time, respectively. To increase the measurement reliability, the gait parameters recorded in two trials of each walking condition were averaged for statistical analysis. In addition, to quantify the interference of additional task on walking, the dual task cost of gait speed (DTC) was calculated according to the following formula. The higher DTC value indicates the more interference caused by the additional task to walking speed.

$${\text{DTC}} = \frac{{s_{ST} - s_{DT} }}{{s_{ST} }} \times 100{{\% }}$$

DTC: dual task cost, S_ST_: walking speed during single walking, S_DT_: walking speed during cognitive (motor) dual task walking.

### Brain activation

A multichannel wearable functional near-infrared spectroscopy (fNIRS) imaging system (NIRSport, NIRx Medical Technologies LLC, Glen Head, NY, USA) was used to detect the cortical hemodynamic response to indicate the brain activation during walking. A total of 14 source-detector channels were arranged over the bilateral prefrontal cortex (PFC), premotor cortex (PMC), and supplementary motor cortex (SMA). The procedure of signal processing referred to our previous study^1^. The data was band-filtered (low-cutoff frequency 0.005 Hz, high-cutoff frequency 0.03 Hz), and wavelet filtering was used for the motion artifact correction in each channel. The preprocessed signals were converted to concentration in oxygenated (HbO) and deoxygenated hemoglobin (HbR) using the modified Beer–Lambert law^56–58^. The relative changes in HbO and HbR concentrations for each walking condition were obtained using a 5-s baseline collected before each session. The HbO and HbR changes were averaged over two repetitions for each walking condition (5–40 s after the onset) to improve signal-to-noise ratio. The index of hemoglobin differential was calculated by (Hbdiff = HbO − HbR) for further analysis. The Hbdiff values in multiple channels corresponding to each brain area were averaged to provide a better indication of hemodynamic response in the specific area (Supplementary Fig. 1). The fNIRS signals were processed using the HOMER2 package, and the calculation of the noise ratio and the Hbdiff values were performed using customized scripts developed on MATLAB (Mathworks, Natick, MA, USA).

### Cognitive function

The cognitive function was assessed by the modified trail making test (TMT) and digit span test.

The TMT are widely used to evaluate executive function in different populations^59^. The modified version of TMT-A and B for the Chinese speakers were used in this study^60^. The time needed to complete each task was recorded for statistical analysis.

Digit span test was used to assess sustained attention and working memory^61^. Participants were asked to repeat series of numbers forward and backward instructed by the assessor. The total scores ranged from 0 to 28, and the higher scores indicated the better performance.

### Interventions

Participants received 24 sessions of 45-min one-on-one training program according to their group assignment.

Participants in CDTT group practiced cognitive tasks during walking on level surface. The cognitive tasks included repeating phrases, counting number forward or backward, playing phonemic word chain games, reciting a poem, having conversations, and reciting a sentence backward.

Participants in MDTT group practiced motor tasks during walking on level surface. The motor tasks consisted of holding balls, raising an umbrella by both hands, waving a rattle, beating a castanet, bouncing a basketball, and holding one ball and concurrently kicking a basketball.

The complexity of walking for both dual-task training groups was modified by walking forward, backward and on an S-shaped route. The difficulty in tasks and walking complexity was progressively adjusted to challenge participants. The 8-week programs for the cognitive and motor dual-task training groups are shown in Supplementary Table 1.

Participants in CPT group received a multicomponent exercise program consisting of muscle strengthening, balance, and gait training for 15 min each. The strengthening exercise focused on the lower extremity muscles, including gluteal muscles, quadriceps, hamstrings, calf muscles and ankle dorsiflexors. The balance training included weight shifting to different directions while standing, tandem standing, standing on a foam, and one-leg standing. Participants were asked to walk forward, backward and on an S-shaped route during the gait training. The resistance for muscle strengthening, and the difficulty of the balance and gait training were adjusted accordingly as the participants progressed.

### Statistical analysis

Non-parametric test was used for comparisons due to heterogeneity of the study sample tested by Shapiro–Wilk test. Intention-to-treat analysis was used for the missing data at follow-up assessment. Descriptive statistics were generated and presented in median (interquartile, IQR) for all variables. The between-group differences in basic data and outcome measures at pre-test were examined using Kruskal–Wallis test and Chi-square test. The within-group training effect on gait performance and cognitive function was evaluated by Friedman test in each group. The between-group differences were analyzed by Kruskal–Wallis test using change values, and post hoc analysis with Dunn–Bonferroni test. Epsilon-square estimate (*ε*^2^) of effect size was calculated for the between-group comparisons (*ε*^2^ = 0.02 as small, 0.13 as medium and 0.26 as large effect size)^62^. Change values were calculated by subtracting the pre-test data from the post-test or follow-up data. Spearman’s rank correlation coefficient was used to determine the correlation between the post-intervention changes in gait and brain activation during walking. All the data was analyzed by SPSS 24.0. The statistically significant level was set at 0.05.

## Supplementary Information

Supplementary Information.

## Data Availability

The datasets generated and analyzed during the current study are available from the corresponding author on reasonable request.
