# Supplementary material for: Effects of different dual task training on dual task walking and responding brain activation in older adults with mild cognitive impairment
Source: Sci Rep. 2022 May 19;12:8490. doi: 10.1038/s41598-022-11489-x (PMC9120469; doi:10.1038/s41598-022-11489-x)
Supplement: Supplementary file 1 — Supplementary Information. [file 41598_2022_11489_MOESM1_ESM.pdf]

Effects of different dual task training on dual task walking and responding brain activation in older adults with mild cognitive impairment

Hsiang-Tsen Kuo, MS[1], Nai-Chen Yeh, MS[2] (co-first), Yea-Ru Yang, PhD[2], Wen-Chi Hsu, MS[3], Ying-Yi Liao, PhD[4], Ray-Yau Wang[2*], PhD

[1]Department of Physical Medicine and Rehabilitation, Taipei Chang Gung Memorial Hospital, Taipei, Taiwan

[2]Department of Physical Therapy and Assistive Technology, National Yang Ming Chiao Tung University, Taipei, Taiwan

[3]Department of Physical Medicine and Rehabilitation, Kaohsiung Municipal United Hospital, Kaohsiung, Taiwan

[4]Department of Gerontological Health Care, National Taipei University of Nursing and Health Sciences, Taipei, Taiwan

Supplementary Figure 1. Arrangement of fNIRS optodes on EEG 10/20 system

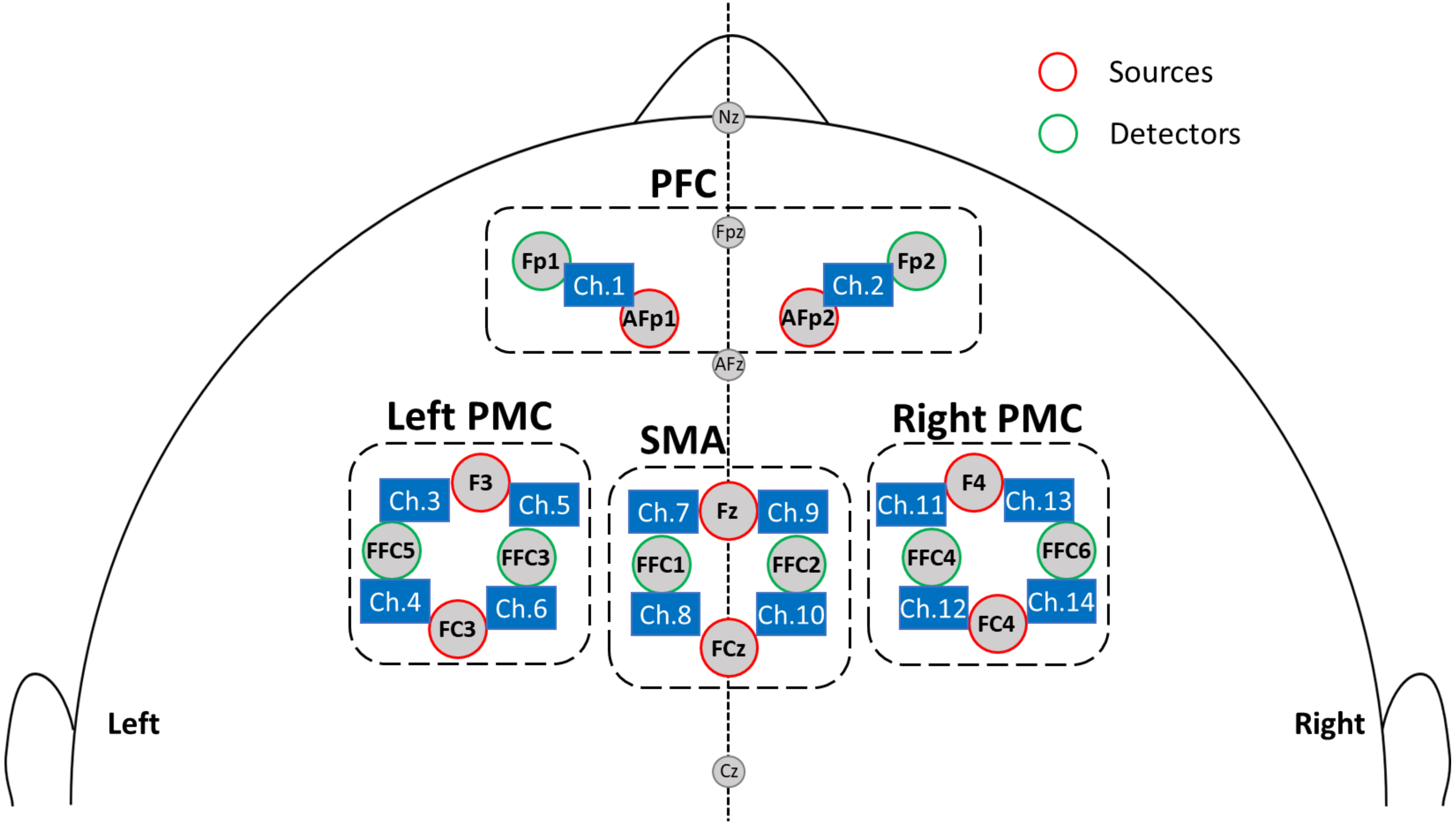

Abbreviations: PFC, prefrontal cortex; SMA, supplementary area; PMC, premotor cortex

Supplementary Table 1. The progression programs for dual task training

Sessions	Walking conditions	Cognitive dual task training / Motor dual task training (mins)						
		Having conversations / Holding a ball/balls	Repeating phrases / Raising an umbrella	Counting number Forward / Waving a rattle	Counting number backward / Beating a castanet	Reciting a sentence backward / Bouncing a basketball	Playing phonemic word chain games / Kicking a basketball	Reciting a poem / Holding one ball with both hands while kicking a basketball
1	Forward	10	10	10	10			
	S shape	5						
2	Forward	5	10	10	10			
	S shape	5	5					
3	Forward	5	5	10	10			
	S shape	5	5	5				
4	Forward	5	5	5	5			
	S shape	10	5	5	5			
5	Forward		5	5	5			
	S shape	5	10	10	5			
6	Forward		5	5	5			
	S shape	5	5	10	10			
7	Forward			10	10			
	S shape		10	5	5			
	Backward	5						
8	Forward			5	5			
	S shape		10	10	10			
	Backward	5						

Supplemental Table 1. The progression programs for dual task training (continued)

Sessions	Walking conditions	Cognitive dual task training / Motor dual task training (mins)						
		Having conversations / Holding a ball/balls	Repeating phrases / Raising an umbrella	Counting number Forward / Waving a rattle	Counting number backward / Beating a castanet	Reciting a sentence backward / Bouncing a basketball	Playing phonemic word chain games / Kicking a basketball	Reciting a poem / Holding one ball with both hands while kicking a basketball
9	Forward			10	10			
	S shape		5	5	10			
	Backward	5						
10	Forward					15	10	
	S shape					5	5	
	Backward			5	5			
11	Forward					15	15	
	S shape					5		
	Backward			5	5			
12	Forward					10	15	
	S shape					5	5	
	Backward			5	5			
13	Forward					10	10	10
	S shape					5		
	Backward			5	5			
14	Forward					10	10	10
	S shape					5	5	
	Backward				5			

Supplemental Table 1. The progression programs for dual task training (continued)

Sessions	Walking conditions	Cognitive dual task training / Motor dual task training (mins)						
		Having conversations / Holding a ball/balls	Repeating phrases / Raising an umbrella	Counting number Forward / Waving a rattle	Counting number backward / Beating a castanet	Reciting a sentence backward / Bouncing a basketball	Playing phonemic word chain games / Kicking a basketball	Reciting a poem / Holding one ball with both hands while kicking a basketball
15	Forward					10	10	15
	S shape					5	5	
16	Forward					10	10	10
	S shape					5	5	5
17	Forward						15	15
	S shape					10	5	
18	Forward						15	15
	S shape					5	5	5
19	Forward					10	10	10
	S shape						10	5
20	Forward					10	10	10
	S shape						5	10
21	Forward						15	15
	S shape					5	5	5
22	Forward					10	10	10
	S shape					5	5	15
23	Forward					5	15	10
	S shape					5	5	5
24	Forward					5	10	15
	S shape					5	5	5

Supplemental table 2. Correlation analysis for the changes in brain activation and gait performance in both dual task training groups

		PFC	Left PMC	Right PMC	SMA
**Cognitive dual task training group (n=9)**					
**SW**	speed	0.12	0.14	0.29	0.43
	Cadence	0.24	0.26	0.21	0.57
	Stride length	0.26	0.29	0.43	0.02
	Stride time	-0.29	-0.36	-0.19	-0.57
**CDW**	speed	-0.02	-0.19	-0.12	-0.17
	Cadence	-0.21	-0.38	-0.24	-0.24
	Stride length	0.85*	0.78*	0.84*	0.91*
	Stride time	-0.143	0.048	0.000	-0.143
**MDW**	Cadence	0.81*	0.57	0.71*	0.43
	stride time	-0.87*	-0.64	-0.93*	-0.67
	DTC	-0.31	-0.26	-0.24	-0.19
**Motor dual task training group (n=11)**					
**SW**	speed	0.19	0.16	-0.03	0.48
	cadence	0.33	-0.09	0.08	0.43
	stride length	0.19	0.24	-0.14	0.42
	spatial v.	0.40	0.10	0.12	-0.01
	stride time	-0.35	0.10	-0.10	-0.43
**CDW**	speed	-0.40	0.05	-0.03	0.05
	cadence	-0.29	-0.03	0.04	-0.12
	stride length	-0.40	0.08	-0.02	0.08
	spatial v.	0.55	0.07	0.01	0.12
	stride time	0.31	0.02	-0.01	0.18
	DTC	0.55	0.07	0.32	0.33
**MDW**	speed	-0.02	-0.08	-0.44	0.27
	cadence	0.26	-0.27	-0.25	-0.03
	stride length	-0.07	0.09	-0.47	0.32
	spatial v.	0.86*	0.58	0.45	0.58
	stride time	-0.26	0.46	0.30	0.13
	DTC	-0.64	-0.03	-0.21	-0.35

Values are Spearman's *rho*

*$p<0.05$ as significant correlation

Abbreviations: SW, single walking; CDW, cognitive dual task walking; MDW, motor dual task walking; PFC, prefrontal cortex; PMC, premotor cortex; SMA, supplemental motor area
